# Cystocerebral Syndrome: An Updated Review and a New Proposed Mechanism for an Often Forgotten Cause of Delirium

**DOI:** 10.7759/cureus.11034

**Published:** 2020-10-19

**Authors:** Franklin L Thelmo, Stephanie Tzarnas, Nathaniel R Rosal, Mackenzie Kramer, Laura Walters

**Affiliations:** 1 Medicine, Abington Memorial Hospital - Jefferson Health, Abington, USA

**Keywords:** cystocerebral syndrome, delirium, urinary obstruction, geriatrics, confusion

## Abstract

Cystocerebral syndrome is an often forgotten cause of delirium in elderly males, which is quite easily treated. We reviewed the current body of literature documenting cystocerebral syndrome and proposed a new mechanism of action explaining why all patients identified thus far have been male.

Data was obtained from articles describing cases of cystocerebral syndrome, urinary retention, and confusion in addition to delirium via a PubMed database search. We reviewed all articles describing cases of cystocerebral syndrome via the PubMed database using the Medical Subject Headings (MeSH) keywords of "cystocerebral syndrome,” urinary retention and confusion," and "delirium and urinary retention or cystocerebral syndrome," and identified eight cases of cystocerebral syndrome including the original publication by Blackburn and Dunn.

We found that all patients reported in the literature were males older than 70 years and often with concomitant benign prostatic hypertrophy (BPH) who presented with acute episodes of delirium that rapidly responded to bladder decompression.

The authors seek to update the medical community regarding this uncommon phenomenon of delirium in elderly male patients. We also propose that the lack of female patients in the literature is reflective of their decreased intraurethral flow resistance as is currently being described in other avenues of research in the field of urodynamics.

## Introduction and background

Delirium is a term that is often used interchangeably with altered mental status, encephalopathy, and a confusional state. According to the Diagnostic and Statistical Manual of Mental Disorders, Fifth Edition (DSM-5) criteria, delirium is an acute disturbance in attention, cognition, perception, and/or language from a patient’s baseline directly due to another medical condition [1]. Etiologies are numerous and can include infections, metabolic derangements, ingestions of drugs or toxins, and even hospitalization. Urinary tract infections, hyponatremia, and uremia have long been established as common causes of delirium in the elderly population, while alcohol and illicit drugs are common causes in young and middle-aged adults. Often, delirium resolves upon the correction of the underlying medical condition. Herein we delve into a rather easily reversible yet often forgotten cause of delirium: cystocerebral syndrome.

## Review

What is cystocerebral syndrome?

Cystocerebral syndrome is broadly defined as encephalopathy or delirious state with features of agitation, paranoia, confusion, decreased responsiveness, and difficulty with redirection in the setting of bladder distention [2,3]. The syndrome was first identified by Blackburn and Dunn in 1990 in a case series of three male patients over the age of 70 who developed features of delirium. These patients had rapid neuropsychiatric recovery upon the insertion of a urinary catheter with decompression of their distended bladder. Since then, the syndrome has been anecdotally identified in several other patients: always male, older than 70 years, and often with a history of benign prostatic hypertrophy (BPH). A number of case reports have since been published on cystocerebral syndrome; however, extensive reviews of the literature are scarce.

Hypothesized pathophysiology

The mechanism behind cystocerebral syndrome is hypothesized to be an acute metabolic encephalopathy from excessive sympathetic drive. This sympathetic drive not only stems from bladder distention. Bladder distention occurs as a result of outlet obstruction most frequently due to BPH followed by significant neuropathy and constipation [3]. Though micturition normally occurs via the parasympathetic nervous system, the sympathetic nervous system via the inferior hypogastric plexus allows the detrusor muscle of the bladder to relax and accommodate excess urine [4]. As the bladder outlet pressures remain elevated, the detrusor muscle attempts to continue relaxing via the upregulation of peripheral receptor β-activation from the sympathetic nervous system. However, this excess catecholamine surge leads to encephalopathy, with features similar to patients experiencing a significant stressor such as in sepsis [4,5]. This explanation most strongly corroborates the clinical presentation of patients with cystocerebral syndrome. Although there remains heterogeneity among presentations in terms of tachycardia and hypertension, this can be explained as a blunting of the response to catecholamines in the elderly population. Additionally, this population has increased the use of antihypertensives and neurohormonal modulators to mitigate adverse cardiac events.

Increased rates of bacterial colonization in the elderly can contribute to small amounts of inflammation of the bladder wall leading to further release of stress hormones as the amount of time the urine spends in the bladder is increased. Furthermore, the inability to micturate leads to excess stimulation of the locus coeruleus, which has been demonstrated to result in hyperarousal [6]. This feed-forward cycle ultimately leads to an elevation of stress metabolites causing patients to become confused, agitated, paranoid, and disoriented. The likelihood of such an event is heightened in elderly patients given their advanced age, leading to decrement in cognitive activity and the ability to cogitate in settings of high stress.

Hypothesis Regarding Affliction of Male Patients

Male patients appear to be uniquely affected by this phenomenon as is evidenced by our literature review. This is most likely due to cystocerebral syndrome being the result of increased intraurethral pressures in the setting of BPH. We also hypothesize that in cases where BPH was not found to be a significant culprit yet only male patients were afflicted, increased length of the urethra in male patients must have led to a degree of resistance resulting in continued retention [7]. The average male urethra is 18-20 cm in length versus the average female urethra of 3-4 cm in length. Accounting for insignificant differences in urine viscosity, this leads to a resistance of flow 5-6x greater in males compared to females in accordance with fluid dynamics principles of the Hagen-Poiseuille equation (Equation 1). This concept is further supported by current research, such as that of Yang et al, regarding pressure-flow studies in the field of urodynamics [8,9].

Equation 1: here “R” is representative of resistance to flow and is related to the inherent viscosity of the fluid “η” and length of tube “L” and inversely against “π” and the “r” radius of the tube.

$$R=8\eta L/(\pi r^4 )$$

Literature review

On July 8, 2020, a literature review was performed of English-language articles via PubMed with the following Medical Subject Headings (MeSH) keywords: “cystocerebral syndrome”, urinary retention and confusion”, and “delirium and urinary retention or cystocerebral syndrome” (Table 1). It yielded 97 articles after the removal of non-English language articles, articles not relevant to search criteria, and redundancies; six articles documenting recognized cases of cystocerebral syndrome were identified (Table 1). Of these six articles, eight cases involved only men with ages ranging from 71 to 88 years. Of these patients, 50% had a history of either BPH or episodes of urinary retention, with one patient having a prior transurethral resection of the prostate (TURP). One patient was on the alpha1 antagonist tamsulosin for his BPH prior to the admission. All patients displayed altered sensorium and three patients presented with symptoms for which a urinary process was thought to be at play. All but one patient was able to receive immediate bladder decompression via insertion of an intraurethral catheter, and one required a suprapubic catheter placement followed later by intraurethral catheterization.

Most cases discussed the resolution of a patient's mental status within a few hours, though one case documented noticeable improvement within 15 minutes. Urinary catheters were left in for an average of five days prior to a trial of void. Those with a known diagnosis of BPH were started on an alpha-adrenergic receptor blocker. Treatment with 5-alpha reductase inhibitors proved to be successful in treating acute urinary retention (AUR); however, they were not effective in preventing reinsertion of a Foley catheter after a failed trial of void [10]. Patients with repeated failed trials of voiding were educated on how to self-catheterize intermittently [11]. Indwelling urinary catheters were avoided in all reported patients. At most, patients were either taught as to how to self-catheterize or were intermittently catheterized with assistance. The benefits of chronic indwelling urinary catheters did not outweigh their risks in an elderly patient and were thus avoided in all circumstances. Upon discharge, two patients underwent TURPs for the full resolution of their urinary retention. One patient who, prior to admission, was placed on amitriptyline for depression had his medication discontinued. Only three patients required prolonged use of Foley catheters after discharge, and one of them required a TURP. No patient was discharged on any additional medication to mitigate the risk of recurrent cystocerebral syndrome. There was no further documentation of recurrent cystocerebral syndrome among patients who had any follow-ups post-discharge.

**Table 1:** PubMed literature review y: years-old; M: male; CHF: congestive heart failure; BPH: benign prostatic hypertrophy; HTN: hypertension; Abx: antibiotics; AML: acute myeloid leukemia; BPD: bipolar disorder; s/p: status-post; TURP: transurethral resection of the prostate

Authors	Patient age/sex	Comorbidities	Presentation	Intervention	Outcome
Blackburn and Dunn [2]	1: 72 y/M; 2: 74 y/M; 3: 71 y/M	1: Alzheimer’s; 2: complete heart block; 3: CHF, systemic amyloidosis	1: tremulous, confused, poverty of speech, unable to follow commands, abdominal tenderness; 2: fatigue, weakness, 3rd-degree heart block, dyspnea; 3: confused, tremors, echolalia, suprapubic tenderness	1: bladder catheterization; 2: transvenous pacemaker and bladder catheterization; 3: bladder catheterization followed by TURP	1: rapid resolution of symptoms without recollection of events; 2: rapid resolution of symptoms without recollection of events; 3: rapid resolution of symptoms without recollection of events
Shirvani and Jimenez [3]	79 y/M	BPH, HTN	Agitation, paranoia, UTI	Bladder catheterization, empiric Abx	Calm and cooperative behavior following bladder decompression
Washco, Engel, Smith, and McCarron [10]	83 y/M	AML, BPD, BPH	Agitation, confusion, leg edema	Bladder catheterization	Resolution of mental status
Saga, Kuriyama, Kiwata, and Kimura [11]	87 y/M	Poorly-controlled diabetes mellitus	Delirium, abdominal distention	Bladder catheterization	Resolution of mental status and abdominal distention
Waardenburg [12]	86 y/M	Alzheimer’s, constipation, BPH s/p TURP	Depression followed by unresponsiveness, suprapubic distention	Bladder catheterization	Resolution of mental status
Blè et al. [13]	88 y/M	HTN, urinary retention bouts	Weakness, disorientation, slowed speech, slowed movement, agitation	Suprapubic catheterization	Resolution of mental status

Approach to management

Male individuals for whom there is a concern for cystocerebral syndrome should be evaluated for prior history of urinary retention, new medications, BPH, diabetes mellitus, constipation, and/or concerns for decreasing micturition. The timing of their altered mentation should be considered to ensure no other stressors are at play. Patients should have their suprapubic region palpated to identify signs of tenderness. If available, a bladder scan should be performed to identify the degree of AUR, though this may be less reliable in patients who have an obese body habitus or ascites. Palpation of the prostate via digital rectal exam will identify if BPH is a primary contributor along with a calculation of the International Prostate Symptom Score (IPSS) and urinalysis. If suspicion for AUR is high, immediate bladder decompression should be performed as long as there are no contraindications upon the inspection of the urethral meatus. These patients may receive a one-time catheterization, but given documentation of greater than 1 liter of urine being removed in many patients, it may be easiest to directly progress to Foley catheter insertion [7]. For some patients, a coudé tip catheter might be necessary. If BPH is felt to be the root cause and medical management is deemed appropriate, an alpha-adrenergic receptor antagonist should be initiated at the time of presentation [14,15]. Treatment with 5-alpha reductase inhibitors can also be used to decrease the incidence of AUR in cases not caused by BPH. For patients in whom chronic bladder outlet obstruction persists, a discussion should be had with urology regarding the appropriateness of regular self-catheterizations, medical management, or surgical intervention.

## Conclusions

Cystocerebral syndrome demonstrates a unique etiology of delirium, predominantly among elderly male patient populations, and it is proposed to be due to metabolic encephalopathy from excessive sympathetic drive in the setting of a bladder unable to self-diurese for a variety of reasons. The concern for cystocerebral syndrome is often secondary in a clinician’s evaluation given the need to rule out other, more common acute causes of delirium such as metabolic derangements, toxic ingestions, medication adverse events, or strokes. However, the evaluating physician should be mindful to include this possible syndrome, since the ability to evaluate and treat it as the underlying cause of delirium has been demonstrated to be quick, easy, and relatively inexpensive. We hope that this article will allow physicians to place this condition at the forefront of their minds during evaluation and that further investigations will continue into why such an acute condition strikes without warning.
